# Avoiding late preterm deliveries to reduce neonatal complications: an 11-year cohort study

**DOI:** 10.1186/s12884-017-1650-8

**Published:** 2018-01-08

**Authors:** Noémie Bouchet, Angèle Gayet-Ageron, Marina Lumbreras Areta, Riccardo Erennio Pfister, Begoña Martinez de Tejada

**Affiliations:** 1 Obstetrics Unit, Department of Obstetrics and Gynecology, Geneva University Hospitals and Faculty of Medicine, 30 Boulevard de la Cluse, 1205 Geneva, Switzerland; 2 Clinical Research Centre and Division of Clinical Epidemiology, Department of Community Health and Medicine, Geneva University Hospitals and Faculty of Medicine, 6 rue Gabrielle-Perret-Gentil, 1205 Geneva, Switzerland; 3 Neonatology Unit, Department of Pediatrics, Geneva University Hospitals and Faculty of Medicine, 30 Boulevard de la Cluse, 1205 Geneva, Switzerland

**Keywords:** Late preterm birth, Delivery, Spontaneous, Non-spontaneous, Evidence-based, Trends, Neonatal complications

## Abstract

**Background:**

Late preterm (LPT) newborns, defined as those born between 34 0/7 and 36 6/7 gestational weeks, have higher short- and long-term morbidity and mortality than term infants (≥37 weeks). A categorization to justify a non-spontaneous LPT delivery has been proposed to distinguish evidence-based from non-evidence-based criteria. This study aims to describe rates and temporal trends of non-spontaneous LPT neonates delivered according to evidence-based or non-evidence-based criteria and to evaluate the number of avoidable LPT deliveries, including severe neonatal morbidity rates and associated risk factors.

**Methods:**

Retrospective cohort study including all LPT neonates born at a Swiss university maternity unit between January 1, 2002 and December 31, 2012. Trends of LPT neonates and neonatal complications were assessed across time using Poisson regression and risk factors for neonatal complications by logistic regression.

**Results:**

Among 40,609 singleton live births, 4223 (10.5%) were preterm and 2017 (4.9%) LPT. In the latter group, 26.2% were non-spontaneous (evidence-based: 12.0%; non-evidence-based: 14.2%). The most frequent indications for evidence-based non-spontaneous LPT delivery were severe preeclampsia (51.8%) and abnormal fetal tracing (24.7%). Indications for non-evidence-based non-spontaneous LPT deliveries were hemorrhage (36.2%) and mild preeclampsia (15.7%). LPT birth rates remained stable over time. The rate of neonatal complications after non-evidence-based LPT birth remained high over time (43.8% vs. 43.5% in 2002 and 2012, respectively; *P* = 0.645), whereas the annual proportion of neonatal complications overall showed a decreasing trend (from 38.0% in 2002 to 33.5% in 2012; *P* = 0.051).

**Conclusions:**

LPT birth rates were stable over time, but neonatal complications remained high, particularly after non-evidence-indicated LPT birth. A total of 287 LPT births could have been potentially avoided if an evidence-based protocol for delivery indications had been used. Efforts should be made to avoid non-spontaneous LPT births in order to reduce neonatal complications.

## Background

Since the 1990s, the worldwide rate of preterm births (birth before 37 weeks’ gestation) has been increasing and even represented up to 11% of all live births in 2010 in Europe and the USA [1, 2]. This trend is partly correlated with the rise of medically-indicated preterm delivery in order to reduce stillbirth [3, 4]. Preterm delivery may occur as a result of spontaneous preterm labor, including the preterm premature rupture of membranes (PPROM) or medical interventions, such as labor induction or elective cesarean, that are initiated to reduce poor outcomes associated with specific maternal or fetal conditions [5–7]. Late preterm (LPT) birth, defined as delivery between 34 0/7 and 36 6/7 weeks’ gestation, represents two-thirds of all preterm births and impacts heavily on the rise of medically- indicated deliveries [8–11]. LPT neonates have long been wrongly considered as “near term”. Beliefs about supposed “almost maturity” and the fear of stillbirth motivated weak indications for induced deliveries, such as isolated oligohydramnios or gestational hypertension [12–14].

It is now well established that LPT infants have an increased risk of morbidity and mortality compared to those born at term [15]. In the short term, they have a higher risk than term infants to suffer from respiratory distress syndrome, apnea, hypothermia, hypoglycemia, jaundice, hyperbilirubinemia, necrotizing enterocolitis and intraventricular hemorrhage [14, 16, 17]. This leads to frequent admissions to the neonatal intensive care unit (NICU) and a longer duration of hospitalization with high economic costs [18–20]. In the long term, LPT newborns appear to have an increased risk of cerebral palsy and mental retardation, as well as more behavioral abnormalities than their term peers [21, 22]. Learning disabilities and a lower socioeconomic level than their parents have also been described [23, 24]. Moreover, LPT birth and its consequences have a negative emotional and psychosocial impact on parents and families, which can last well beyond the initial period of hospitalization [25, 26].

Therefore, it is of crucial importance to determine the optimal time of delivery in order to reduce perinatal morbidity and mortality, while balancing neonatal and infant risks [27, 28]. Based on a review of the guidelines of the American College of Obstetricians and Gynecologists’ [29], the obstetrics literature and published expert opinion [27], Gyamfi-Banneman et al. proposed a set of evidence-based (EB) indications to justify a LPT delivery [30, 31]. In the absence of sufficient scientific evidence, indications were considered weak to justify iatrogenic LPT delivery and were thus categorized as non-EB and included elective (non-medical) indications [13].

The aims of this study were to describe the trend of LPT births and their indications over an 11-year period according to the Gyamfi-Banneman categorization [31]. We also sought to assess neonatal complications related to LPT birth and the accompanying risk factors.

## Methods

This retrospective cohort study was conducted at the maternity unit of Geneva University Hospitals, Geneva, Switzerland, and included all LPT births between January 1, 2002 and December 31, 2012. The local institutional ethics committee approved the research protocol.

### Study population

The maternity unit is the largest in Switzerland with approximately 4000 deliveries per year, of which approximately 10% are preterm. We included all births of singletons between 34 + 0 and 36 + 6 weeks’ gestation. Stillbirths and multiple gestations were excluded. We obtained the list of all newborns during the study period from the labor and delivery suite database and gathered maternal and neonatal data from the medical charts using a standardized report form. Relevant data were extracted from the following sources: the maternal and neonatal databases of the obstetrics service and the neonatal database of the pediatric department. All data were coded using a unique study number.

### Outcomes

The primary outcome was the number of LPT births among spontaneous LPT, EB non-spontaneous LPT and non-EB non-spontaneous LPT deliveries. The secondary outcome was an adverse neonatal event defined by the presence of at least one of the following complications: neonatal death; NICU admission; need for ventilatory support; neonatal sepsis with bacteremia; and respiratory disease requiring oxygen or ventilatory support (composite outcome).

### Variables

Data were described on an annual basis. We extracted the following maternal and obstetrical variables: maternal age; gestational age at delivery (based on the first trimester ultrasound); gravidity; parity; history and type of cesarean section; prior myomectomy; chronic hypertension; gestational hypertension; preeclampsia; cholestasis; PPROM; intrauterine growth retardation; abnormal fetal Doppler (umbilical and/or cerebral); abnormal fetal tracing; oligohydramnios; pulmonary maturation; pre-labor uterine rupture; delivery onset: spontaneous or non-spontaneous (labor induction or elective cesarean); indication for delivery; mode of delivery (vaginal delivery with or without instruments, elective or in-labor cesarean section); fetal presentation; and type of anesthesia.

Neonatal variables included gender; birth weight in grams; Apgar score at 5 min; umbilical arterial pH; growth retardation (<10th percentile growth for gestational age); use of ventilatory support by either non-invasive ventilation (continuous positive airway pressure) or invasive ventilation (intubation and mechanical ventilation); duration of hospitalization in days; hospitalization site (maternity, NICU); presence of respiratory pathologies (wet lung, respiratory distress syndrome); neonatal sepsis with positive bacteremia; neonatal malformation; chromosomal abnormalities; and neonatal death at less than 1 month.

We classified LPT birth as either spontaneous or non-spontaneous LPT. Spontaneous LPT birth was defined as the spontaneous onset of uterine contractions and cervical dilation. Women with PPROM and no other indication were included in this group. The non-spontaneous LPT birth group included women with induction of labor or elective cesarean section without contractions. This group was further subcategorized according to Gyamfi-Banneman et al. as either EB or non-EB management. Indications labeled EB were supported by strong scientific evidence based on the guidelines of the American College of Obstetricians and Gynecologists’ [29], the obstetrics literature and published expert opinion [27]. EB indications for non-spontaneous LPT birth were severe preeclampsia or eclampsia, intrauterine growth retardation with abnormal testing (abnormal fetal Doppler or fetal heart tracing, oligohydramnios) or poor interval growth, acute abruption, non-reassuring fetal heart rate tracing, cholestasis, and uterine rupture prior to spontaneous initiation of labor. Indications labeled non-EB were not supported by sufficient scientific evidence and thus weaker to justify a non-spontaneous LPT delivery. Non-EB indications for LPT birth included chronic or gestational hypertension, mild preeclampsia, intrauterine growth retardation with normal testing and adequate interval growth, prior myomectomy or classic cesarean section, isolated oligohydramnios. Any other indication reviewed in medical records and not listed previously was classified as an elective delivery and categorized as non-EB LPT. These indications were in general debated within the clinical team and not supported by scientific evidence, but mainly explained by the wishes of the patient or care provider. In Gyamfi-Banneman et al., only cholestasis with bile acids >40 micromol/L was considered as EB. Cholestasis with bile acids <40 micromol/L were considered as non-EB. In our study, all women with cholestasis were included in the EB group as we did not have measurements of bile acid levels.

### Statistical analysis

All continuous variables were described by their mean ± standard deviation, overall median and LPT group. Categorical variables were described by their frequencies, relative proportion overall and LPT group. A comparison of continuous variables between the three groups of LPT deliveries (i.e. between spontaneous LPT and non-spontaneous LPT or between EB and non-EB non-spontaneous LPT) was done using the Kruskal-Wallis test because of skewed distributions. Categorical variables were compared between the groups using either the Chi-square or Fisher’s exact test. The overall number of complications were then stratified on the three groups of LPT and assessed across time using Poisson regression models.

Finally, we assessed the risk factors associated with the occurrence of at least one complication using a logistic regression model where the LPT group was the main predictor. We pre-specified the following variables as important risk factors for neonatal complications: mode of delivery (spontaneous, elective cesarean or cesarean during labor); pulmonary maturation (yes/no); gestational age strata (34 to 34/6 weeks, 35 to 35/6 weeks, and 36 to 36/6 weeks); maternal age strata (<20, 20–29, 30–34, 35–39 and > = 40 years); birth weight strata (<10th, 10th to 50th, 50 to 90th, and >90th percentile); and gender. Logistic regression provided maximum likelihood estimates of the odds ratios (OR) and their 95% confidence intervals (CI). The goodness-of-fit of the model was verified using the Hosmer-Lemeshow test. The amount of variation in the likelihood of neonatal complications explained by the model was indicated by the Cox and Snell *R*^2^. The discriminant capacity of the model was evaluated by the area under the curve assorted with its 95% CI. Statistical significance was defined as a two-sided *P* value of <0.05. Statistical analyses were performed using Stata IC 14 (STATA Corp., College Station, TX).

## Results

**Fig. 1:**
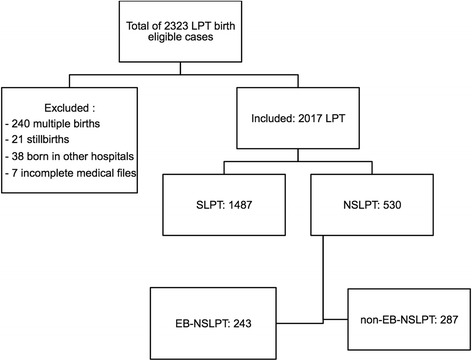
Flow chart of the study population. LPT: late preterm; SLPT: spontaneous late preterm; NSLPT: non-spontaneous late preterm; EB: evidence-based

**Fig. 2:**
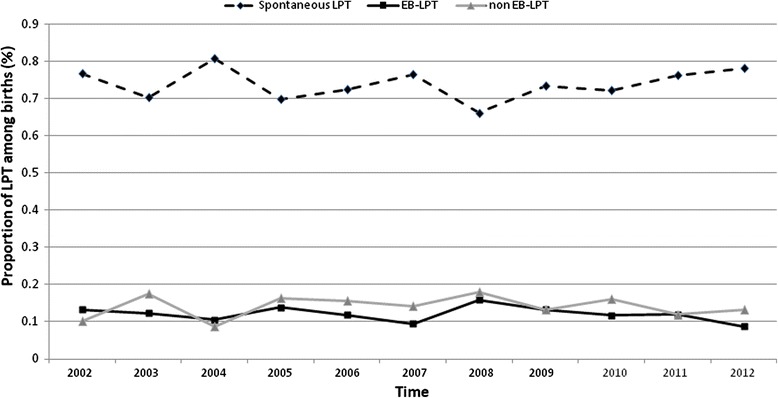
Temporal trends of each type of late preterm birth: Geneva University Hospitals, 2002–2012. LPT: late preterm; EB: evidence-based

A total of 40,609 live singleton deliveries were recorded during the 11-year study period. Among these, 4223 (10.5%) were preterm and 2017 (4.9%) LPT. Among the LPT deliveries, 1487 were classified as spontaneous LPT (73.7%) and 530 (26.2%) as non-spontaneous LPT. In the latter group, 243 (12.0%) were EB and 287 (14.2%) non-EB (Fig. 1). Maternal and obstetric characteristics are shown in Table 1. There were more women with a history of cesarean section (*P* < 0.001) and with chronic or gestational hypertension (*P* < 0.001 in both) in the non-spontaneous LPT than in the spontaneous LPT group. The cesarean section rate was higher in the non-EB non-spontaneous LPT (82.9%) than in the EB group (67.1%; *P* < 0.001). Globally, cesarean section during labor was more frequent in the non-spontaneous LPT group compared to spontaneous LPT cases (OR: 1.8; 95% CI: 1.36–2.39; *P* < 0.001). The proportion of each LPT subgroup remained stable over time (*P* = 0.665 for spontaneous LPT; *P* = 0.532 for EB non-spontaneous LPT; *P* = 0.609 for non-EB non-spontaneous LPT; Fig. 2).

**Table 1:** Overall maternal and obstetric characteristics and type of late preterm delivery

	Overall	Spontaneous LPT (*N* = 1487)	EB-LPT (*N* = 243)	Non EB-LPT (*N* = 287)	*P*-value^a^	*P*-value^b^	*P*-value^c^
Obstetrical description							
Maternal age: mean (±SD, P50)	31.7 (±5.6; 32)	31.4 (±5.5; 32)	32.1 (±5.8; 32)	32.9 (±5.8; 33)	<0.001	0.06	<0.001
Mean gestational age in weeks (±SD, P50)	35.3 (±0.8; 36)	35.4 (±0.8; 36)	35.2 (±0.8; 35)	35.4 (±0.8; 36)	0.002	0.005	0.159
Gravidity, n (%)					<0.001	<0.001	0.625
Primigravida	714 (35.4)	531 (35.7)	110 (45.3)	73 (25.4)			
Multigravida	1303 (64.6)	956 (64.3)	133 (54.7)	214 (74.6)			
Parity, n (%)					<0.001	<0.001	0.399
Primiparous	1063 (52.7)	792 (53.3)	160 (65.8)	111 (38.7)			
Multiparous	954 (47.3)	695 (46.7)	83 (34.2)	176 (61.3)			
Prior caesarean section, n (%)					<0.001	<0.001	<0.001
No	1786 (88.5)	1360 (91.5)	217 (89.3)	209 (72.8)			
Segmental caesarean	213 (10.6)	117 (7.9)	23 (9.5)	73 (25.4)			
Non-segmental caesarean	18 (0.9)	10 (0.7)	3 (1.2)	5 (1.7)			
Prior myomectomy, n (%)	5 (0.3)	2 (0.1)	0 (0)	3 (1.0)	0.013	0.254	0.086
Chronic hypertension, n (%)	28 (1.4)	7 (0.5)	15 (6.2)	6 (2.1)	<0.001	0.016	<0.001
Gestational hypertension, n (%)	63 (3.1)	21 (1.4)	24 (9.9)	18 (6.3)	<0.001	0.126	<0.001
Preeclampsia, n (%)	212 (10.5)	39 (2.6)	128 (52.7)	45 (15.7)	<0.001	<0.001	<0.001
Severe preeclampsia, n (%)	127 (60.5)	0 (0)	127 (100.0)	0 (0)	<0.001	<0.001	<0.001
Cholestasis, n (%)	3 (0.2)	0 (0)	3 (1.2)	0 (0)	<0.001	0.096	0.004
Haemorrhage, n (%)					<0.001	<0.001	<0.001
No	1896 (94.0)	1480 (99.5)	233 (95.9)	183 (63.8)			
Placenta praevia	37 (1.8)	0 (0)	0 (0)	37 (12.9)			
Acute abruption	0 (0)	0 (0)	10 (4.1)	0 (0)			
Other, missing data	74 (3.7)	7 (0.5)	0 (0)	67 (23.3)			
Rupture of membranes, n (%)	1090 (54.0)	1079 (72.6)	43 (17.7)	0 (0)	<0.001	<0.001	<0.001
IUGR, n (%)	143 (7.1)	43 (2.9)	71 (29.2)	29 (10.1)	<0.001	<0.001	<0.001
Abnormal doppler, n (%)	42 (2.1)	0 (0)	36 (14.8)	6 (2.1)	<0.001	<0.001	<0.001
Oligohydramnios, n (%)	79 (3.9)	34 (2.3)	13 (5.4)	32 (11.2)	<0.001	0.019	<0.001
Abnormal fetal tracing, n (%)	73 (3.6)	0 (0)	73 (30.0)	0 (0)	<0.001	<0.001	<0.001
Lung maturation, n (%) (*n* = 1977)	373 (18.5)	220 (14.8)	48 (19.8)	105 (36.6)	<0.001	<0.001	<0.001
Uterine rupture, n (%)	0 (0)	0 (0)	0 (0)	0 (0)	–	–	–
Start of delivery, n (%)					<0.001	<0.001	<0.001
Spontaneous	883 (43.8)	880 (59.2)	3 (1.2)	0 (0)			
Induced	934 (46.3)	564 (37.9)	206 (84.8)	164 (57.1)			
Elective caesarean	200 (9.9)	43 (2.9)	34 (14.0)	123 (42.9)			
Mode of delivery, n (%)					<0.001	<0.001	<0.001
Vaginal	1278 (63.4)	1150 (77.3)	79 (32.5)	49 (17.1)			
Elective caesarean	200 (9.9)	43 (2.9)	34 (14.0)	123 (42.9)			
Cesarean in labour	539 (26.7)	294 (19.8)	130 (53.5)	115 (40.1)			
Presentation, n (%)					<0.001	<0.001	0.008
Cephalic	1325 (89.2)	983 (90.7)	175 (93.1)	167 (78.0)			
Breech	146 (9.8)	92 (8.5)	13 (6.9)	41 (19.2)			
Other	15 (1.0)	9 (0.8)	0 (0)	6 (2.8)			
Type of anaesthesia, n (%)					<0.001	0.111	<0.001
Non	347 (17.2)	332 (22.3)	9 (3.7)	6 (2.1)			
Epidural	1629 (80.8)	1139 (76.6)	227 (93.4)	263 (91.6)			
General	41 (2.0)	16 (1.1)	7 (2.9)	18 (6.3)			
Neonatal description							
Female gender, n (%)	928 (46.0)	643 (43.2)	127 (52.3)	158 (55.1)	<0.001	0.521	<0.001
Birth weight (kg): mean (±SD, P50)	2.57 (±0.46, 2.56)	2.63 (±0.43, 2.61)	2.19 (±0.47, 2.14)	2.55 (±0.45, 2.56)	<0.001	<0.001	<0.001
Apgar <5, n (%)	14 (0.7)	10 (0.7)	2 (0.8)	2 (0.7)	0.966	0.999	0.769
Arterial pH < 7.10, n (%)	67 (3.5)	43 (3.1)	15 (6.4)	9 (3.3)	0.038	0.140	0.094
Growth retardation <p10, n (%)	173 (9.0)	72 (5.0)	77 (35.0)	24 (9.2)	<0.001	<0.001	<0.001
NICU stay, n (%)	828 (41.0)	518 (34.8)	161 (66.3)	149 (51.9)	<0.001	0.001	<0.001
Malformation, n (%)	46 (5.5)	24 (4.6)	7 (4.4)	15 (10.0)	0.033	0.054	0.138
Ventilatory support, n (%)					<0.001	0.428	<0.001
No	1733 (85.9)	1322 (88.9)	194 (79.8)	217 (75.6)			
Non-invasive	246 (12.2)	146 (9.8)	40 (16.5)	60 (20.9)			
Invasive	38 (1.9)	19 (1.3)	9 (3.7)	10 (3.5)			
Mean stay at NICU in days (±SD, P50) (*n* = 828)	12.1 (±14.2, 10)	11.4 (±13.7, 9)	13.3 (±13.4, 11)	13.0 (±16.5, 9)	0.226	0.067	0.006
Neonatal sepsis with bacteremia, n (%)	5 (0.6)	2 (0.4)	2 (1.2)	1 (0.7)	0.458	0.999	0.367
Respiratory diseases, n (%)	419 (21.1)	256 (17.5)	72 (30.6)	91 (32.2)	<0.001	0.711	<0.001
Neonatal death, n (%)	4 (0.2)	1 (0.07)	2 (0.8)	1 (0.35)	0.036	0.596	0.058
Presence of ≥1 complication^*^, n (%) (*n* = 2014)	829 (41.2)	518 (34.9)	161 (66.3)	150 (52.3)	<0.001	0.001	<0.001

*LPT* late preterm, *EB* evidence-based, *SD* standard deviation, *IUGR* intrauterine growth retardation, *NICU* neonatal intensive care unit, *SD* standard deviation

^a^Comparisons between the three groups of LPT birth; ^b^comparisons between spontaneous LPT birth and EB plus non-EB-LPT birth; ^c^comparisons between EB and non-EB-LPT birth

Complication *: neonatal death; NICU admission; need for ventilatory support; neonatal sepsis with bacteremia; and respiratory disease requiring oxygen or ventilatory support

The most frequent indications for EB non-spontaneous LPT were severe preeclampsia (51.8%), abnormal fetal tracing (24.7%) and intrauterine growth retardation with abnormal testing. For non-EB non-spontaneous LPT cases, the most common indications were hemorrhage without placenta abruption (36.2%) and non-severe preeclampsia (15.7%). Elective indications accounted for 34.5% of non-EB non-spontaneous LPT and included various diagnoses, such as iso-immunization, maternal life-threatening conditions (i.e. breast cancer, acute renal failure, maternal sepsis), severe fetal malformation (laparoschisis, major renal defect) and maternal psychological distress related to pregnancy. Few cases were “strictly elective” without any clearly reported medical indication.

**Fig. 3:**
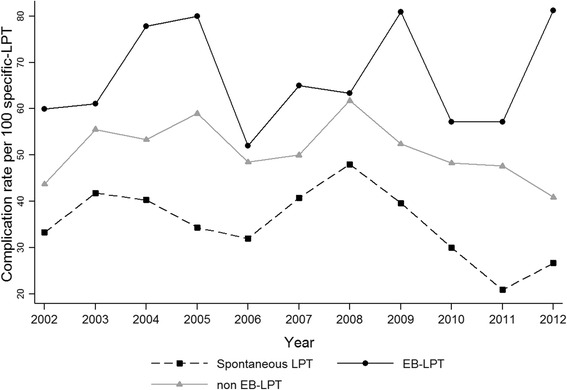
Incidence rates of complications in the three late preterm birth groups. LPT: late preterm; EB: evidence-based

The proportion of infants hospitalized in the NICU was significantly higher in the non-spontaneous LPT compared to the spontaneous LPT group (Table 1). The number of neonatal complications in the three LPT groups decreased over time, but the decrease was only statistically significant in the spontaneous LPT group (*P* = 0.031). The likelihood of neonatal complications was 3.66-fold (95% CI: 2.75–4.87) higher in EB non-spontaneous LPT compared to spontaneous LPT, 2.04-fold higher (95% CI: 1.58–2.63) in non-EB non-spontaneous LPT compared to spontaneous LPT, and 1.79-fold higher (95% CI: 1.26–2.55) in EB non-spontaneous LPT compared to non-EB non-spontaneous LPT (Fig. 3). After adjustment for the main confounders (Table 2), the likelihood of complications remained significantly higher among non-EB non-spontaneous LPT compared to spontaneous LPT cases (*P* < 0.001), but there was no significant difference between non-spontaneous non-EB LPT and EB LPT (*P* = 0.225). There was a trend for higher odds of complications in the non-spontaneous EB LPT group compared to spontaneous LPT. The odds of neonatal complications were also significantly and independently increased in the in-labor cesarean section group compared to vaginal deliveries, in boys compared to girls, and in neonates with a birth weight between the 10th to 50th percentile and below the 10th percentile compared to infants with a birth weight above the 90th percentile. The multivariable model was also adjusted for maternal age and lung maturation and demonstrated a good discriminant capacity with an area under the curve of 0.83 (95% CI: 0.81–0.85).

**Table 2:** Associated factors with the likelihood of neonatal complication (multivariate analysis)^a^

Variables	Odds ratio	95% confidence interval	*P*-value
Time periods (reference 2002–2004)			<0.001
2005–2006	0.83	0.59–1.15	0.258
2007–2008	0.96	0.68–1.36	0.830
2009–2012	0.47	0.34–0.64	<0.001
Group of LPT (reference spontaneous)			0.001
EB-LPT	1.49	0.99–2.22	0.051
Non EB-LPT	2.03	1.37–3.00	<0.001^a^
Mode of delivery (reference spontaneous)			0.0002
Elective caesarean	1.53	0.96–2.45	0.074
Caesarean during labour	1.80	1.36–2.39	<0.001
Lung maturation (reference no lung maturation)	1.01	0.75–1.37	0.941
Gestational age in weeks (reference [36])			<0.001
34	27.3	19.22–38.79	<0.001
35	3.15	2.41–4.10	<0.001
Mother’s age in years (reference <20 years)			0.426
20–30	1.59	0.60–4.21	0.350
30–35	1.47	0.56–3.90	0.435
35–40	1.22	0.46–3.25	0.690
> =40	1.61	0.57–4.56	0.366
Weight percentile categories (reference >90th percentile)			<0.001
50-90th	1.08	0.76–1.53	0.665
10-50th	1.62	1.13–2.31	0.009
10th	16.18	9.46–27.67	<0.001
Boy (reference girl)	1.66	1.31–2.11	<0.001

*LPT* late preterm, *EB* evidence-based

^a^Amount of variation in the likelihood of neonatal complication explained by the model was 29%

## Discussion

Overall, approximately one-quarter of LPT deliveries at our center were iatrogenic. When stratified according to the classification proposed by Gyamfi-Banneman et al..., one-half were for non-EB indications [13]. In contrast to the current trend of increasing LPT delivery rates often attributed to non-spontaneous LPT birth, our rates remained stable over time and were slightly lower than those reported by other authors using the same criteria (17% by Gyamfi-Banneman et al. [13, 31] and 18% by Holland et al) [32]. Morais et al. reported a higher rate of 25.5% non-EB LPT, but included PPROM in this group [33]. By excluding PPROM, the rate decreased to 10.7%, the lowest reported so far.

In our study, the overall rate of neonatal complications in LPT tended to decrease, although the rate after non-EB LPT birth did not change over time. As guidelines for lung maturation during the late preterm period in women at risk of delivery have changed recently, we can expect a decreasing rate of respiratory morbidity in LPT neonates in the future [34, 35]. Nevertheless, newborns born after non-EB LPT management received lung maturation more often than the other two groups and the rate of complications was higher, thus emphasizing the importance of avoiding the occurrence of these cases.

Non-spontaneous LPT newborns require more neonatal care than spontaneous LPT as they are more frequently admitted to the NICU, have a longer hospital stay and require more ventilatory support [14]. The risk persisted after adjustment for independent risk factors of neonatal complications (mode of delivery, pulmonary maturation, gestational age, maternal, birth weight and gender). By strictly avoiding non-EB LPT (14.2% of LPT) deliveries, it might be possible to reduce the burden of neonatal morbidity up to a factor of two. We assume that potential supplementary factors may have influenced the obstetrical decision.

Elective indications accounted for 34.5% of non-EB non-spontaneous LPT deliveries and included various diagnoses, such as maternal life-threatening conditions (i.e. breast cancer, acute renal failure, maternal sepsis), fetal malformation, etc. Although these indications are not considered as EB, their severity could explain the indication for delivery and the poor neonatal outcome [36].

Antepartum hemorrhage was a frequent indication for non-EB non-spontaneous LPT birth and included placenta praevia, vasa praevia, suspicion of uterine rupture and severe genital bleeding of unknown origin. Acute abruption and uterine rupture were considered EB non-spontaneous LPT indications. Placenta praevia indicated delivery between 35 5/7 weeks and 36 6/7 weeks in 37 cases, which is in agreement with recent recommendations from the American College of Obstetricians and Gynecologists Committee on Obstetric Practice [29]. Therefore, we suggest that the categorization of Gyamfi-Banneman et al. should include placenta praevia as an EB indication for delivery during the late preterm period, possibly after 36 0/7 weeks if uncomplicated (no fetal growth restriction, no hemorrhage or repetitive bleeding, or no other additional EB indication). Fifteen cases were delivered due to suspected uterine rupture, although none was confirmed. By excluding the 52 cases (placenta praevia and suspicion of uterine rupture) where we disagree with the indications of Gyamfi-Banneman et al., we estimate that we could have potentially avoided 235 LPT deliveries.

The strengths of the present study are related to the use of the same categorization for LPT delivery as Gyamfi-Banneman et al., thus ensuring reproducible results and allowing to compare practices within institutions nationally and internationally. The study duration allowed to collect a large number of LPT cases (2017) with complete neonatal follow-up in a single center.

Our study has some limitations. First, its observational design means that the effect of unknown confounders could have impacted on the association between the LPT groups and neonatal complications. To account for this potential bias, we adjusted for the main known confounders. Second, we were unable to classify severe cholestasis as we did not measure bile acid and our three cases of cholestasis were classified as EB. Third, cases of PPROM were systematically induced from 34 weeks’ gestation and classified as spontaneous LPT birth. However, based on recent evidence that an expectant management of PPROM provides more benefits without increasing the rate of neonatal sepsis [37], practices related to PPROM during the late preterm period have recently changed in our maternity unit. This subgroup represented quite a large number of cases (1076) and it would now be considered PPROM without signs of complications as a non-EB indication for delivery, similar to Morais et al. [33].

Our aim was not to establish a new, strict and exhaustive classification of EB and non-EB criteria, but rather to support only EB indications. We also aimed to identify ways to reduce the number of non-spontaneous LPT deliveries in our setting and more generally. At present, this categorization is evolving and future research in the field of LPT management may help to improve the definition of EB and non-EB indications. In our setting, our study allowed to highlight that one-half of non-spontaneous LPT births could have been avoided if current recommendations had been applied. When possible, obstetrical settings should be encouraged to use this classification, which aims to improve the management of LPT births. A recent study reported that strengthening management policies of non-EB non-spontaneous LPT indications led to a decline in preterm birth [38]. Another study found that the presence of condition-specific obstetric protocols did not lead to detectable improvements in pregnancy outcomes [39]. Although Clark et al. reported that an association of protocols with a “hard stop” policy was the best way to improve EB medicine practices [40], the necessity for strict protocols in obstetric management remains a subject of debate among specialists.

## Conclusions

The proportion of LPT births remained stable over the entire study period. One-quarter of LPT births were non-spontaneous and more than half of these were non-EB with a high risk of neonatal complications. Between 287 and 235 could have been avoided if strict criteria had been applied. Efforts should continue to reduce unnecessary LPT births in order to reduce neonatal morbidity.

## Abbreviations

CI: Confidence interval
EB: Evidence-based
IUGR: Intrauterine growth retardation
LPT: Late preterm
NICU: Neonatal intensive care unit
OR: Odds ratio
PPROM: Preterm premature rupture of membranes
SLPT: Spontaneous late preterm delivery
